# On What Is Commonly Called “Rigidity of the Os Uteri”

**Published:** 1861-03

**Authors:** Charles D. Arnott

**Affiliations:** Edin., &c.


*On what is commonly called "Rigidity of the Os Uteri.* By CHARLES D. ARNOTT, M. D. Edin., &c.

Amongst the many causes of delay and difficulty in human parturition, abnormal conditions opposing the completion of the preparatory process in the maternal passages for the descent of the fœtus, are of frequent occurrence. Obstetric writers recognize one of these as claiming paramount importance, and describe it under the term, "rigidity of the os uteri."

The following brief outline may serve as an illustration of a case often met with in practice. A patient is in labour with her first child, and has been so many hours. The pains have been regular, have gradually increased in severity, and have now reached such intensity, that the nurse and friends are assured the necessary assistance only is needed to effect speedy delivery. Upon arrival, the medical attendant can but be similarly impressed; the pains recur regularly, are strong, and present all the characters of the genuine expulsive efforts, the last in the train. Digital examination reveals the following conditions:—Vaginal passage dry and tight; os uteri barely within reach, high up posteriorly, a mere slit like a button-hole, scarcely admitting the finger's point; the fœtal head low down anteriorly, pushing before it a large segment of uterine tissue, and under the influence of each pain apparently making ineffectual efforts to force its way through this evident source of obstruction. Under these circumstances, inexpressibly unhappy, the labour, without adequate assistance, must inevitably persist for hours, perhaps even for days.

This is a correct delineation of those cases which are described as depending upon rigidity of the os uteri; but, as I apprehend, improperly so. In the great majority, the os is, in reality, dilatable enough, but some of the chief elements of dilatation are acting disadvantageously, or not at all. The fluid wedge, for instance, is generally absent, the membranes having ruptured early under the influence of the strong uterine contractions, and the fœtal head is expending all its force in a wrong direction, not upon the os uteri, as occurs under normal conditions, but upon that shelf of uterine texture it has infringed upon, and which so long must successfully resist it.

The treatment of these cases the teaching of the schools has hitherto inculcated, I am now fully convinced, is not only inert, but highly injudicious. Bloodletting and antimonial depressants (time-honored maids-of-all-work) have been loudly extolled as adjuvants of vital dilatability, and almost implicitly relied on. Anodynes (learnedly) to lull excessive irritability, (truthfully) a clumsy excuse to gain time, have also had their share of renown, and these again are now likely to be superseded by a more potent agent, chloroform.*

I shall not discuss in full the merits of these plans of treatment. I am, from repeated experience, as fully convinced as I ever can be of any one professional fact, not only that they are of no use whatever, but that they are positively and highly injurious. My opinion is confirmed, that the effectual treatment of these cases is by surgery alone; reduction and dilatation of the abnormally placed os by manipulation, and in the more aggravated and resisting cases, incision of the opposing texture. Much good service may, indeed, thus be rendered, a great amount of maternal suffering saved, and infantile life preserved.

Free lubrication of the parts having been premised, the finger is to be introduced during each pain, and the os solicited downwards and forwards, and at the same time freely dilated without any unjustifiable force. As with the prostate after the deep incision in lithotomy, so with the os in the great majority of these cases, it will be found to yield readily. Improvement commences forthwith, and all difficulty is soon overcome. In the very rare minority, incision (mere notching with a guarded probe-pointed bistoury) may be requisite in addition to manipulation, and the most intractable cases are speedily made to assume a totally different and more promising aspect.

I am aware the proposal seems harsh, and may be denounced by the *laisser aller* school as belonging to that confessedly bad category, "meddlesome" mid-wifery. I believe, however, it may be fully proved undeserving any such epithet, by the remembrance that there are cases which tax our resources to the highest extent, from the extreme anxiety generally pertaining to them, and the inefficacy of the means

---

* See a graphically detailed and characteristic case, published in THE LANCET (Vol. 1, 1860, page 397,) by F. Dumaresque Ross, Esq., Guilford, in which he expresses much satisfaction from its use, although the account shows the labour persisted twenty-five anxious hours after its employment.

usually employed for their relief; and further justified by the great fact of its being merely a close imitation of Nature's own *modus operandi* under these special circumstances.

Let us briefly inquire how this difficulty is ordinarily surmounted by Nature's efforts when unaided? Almost always, as all observant practitioners have again and again experienced, by spontaneous laceration, sometimes rather extensive, of the resisting uterine tissues; and, in spite of bloodletting, antimonials, and all other such auxiliaries, as they are inaccurately termed, scarcely ever, until this is accomplished, can she complete the process. Instances in which very extensive rupture ensues, are, with this concomitant complication, not uncommon; and many so-called cases of occlusion of the os uteri, in which incision is the only alternative, Nature having failed in effecting the necessary laceration, are probably often little more than extreme cases of the description we are now considering, in which the os, being more out of reach than usual, escaped detection.

I have employed the practice now recommended for many years, and on many occasions I have satisfied myself of its invariable and great utility; moreover, having never observed one untoward sequence from its employment, I conclude it may be regarded almost entirely free from danger. Let it but have a fair, unprejudiced trial, and I feel assured it will so forcibly commend itself, as to assert its superiority over all other expedients.—*London Lancet.*

*Gorleston, Great Yarmouth*, 1861.

